# COGNITION TRAJECTORY AND DISABILITY AMONG OLDER AMERICAN COUPLES: THE MEDIATING ROLE OF DEPRESSIVE SYMPTOMS

**DOI:** 10.1093/geroni/igad104.1874

**Published:** 2023-12-21

**Authors:** Dexia Kong, Peiyi Lu, Da Jiang, Helen Chan

**Affiliations:** The Chinese University of Hong Kong, Hong Kong, Hong Kong; Columbia University, New York City, New York, United States; Education University of Hong Kong, Hong Kong, Hong Kong; The Chinese University of Hong Kong, Hong Kong, Hong Kong

## Abstract

**Background:**

Using a dyadic approach, this study examined the mediating effect of depressive symptoms on the longitudinal relationships between husbands’ and wives’ memory trajectories and their prospective disability status. Method: Longitudinal data from Health and Retirement Study 2004-2018 were used. Older (aged 50+) heterosexual couples married for 5+ years and had no limitations on activity of daily living at baseline (2004) were included (N=1,310). Latent class growth analysis grouped wives and husbands into distinct memory trajectories in 2004-2014. Structural Equation Model examined the actor and partner effects of memory trajectories on depressive symptoms in 2016 and disability status in 2018. The mediating effect of depressive symptoms was tested.

**Results:**

Four distinct trajectories were found: persistently high (wives: 11.83%; husbands: 3.83%); high and slow decline (wives: 36.26%; husbands: 33.66%); moderate and slow decline (wives: 41.30%; husband: 47.97%); low and rapid decline (wives: 10.61%; husbands: 14.54%). Only the wife’s low and rapid decline memory trajectory predicted her own more depressive symptoms (β=0.588, 95% CI=0.209, 0.967) and her husband’s more depressive symptoms (β=0.326, 95% CI=0.004, 0.648). Meanwhile, depressive symptoms had strong and significant actor effects on disability (β=0.046, 95% CI=0.036, 0.057 in the wife’s outcome; β=0.060, 95% CI=0.046, 0.074 in the husband’s outcome).

**Conclusions:**

Wife’s low and rapid decline trajectory was associated with her own and her husband’s more depressive symptoms, which in turn increased the disability risk of both partners. Detection and treatment of cognitive decline among wives have the potential to mitigate the couples’ depressive symptoms and ultimately disability risks.

